# Considerations beyond spine pain: do different co-occurring lower body joint pains differentially influence physical function and quality of life ratings?

**DOI:** 10.1186/s12891-024-07393-2

**Published:** 2024-04-08

**Authors:** Shawn McGargill, Michael Sein, Kimberly T. Sibille, Zane Thompson, Michael Brownstein, Heather K. Vincent

**Affiliations:** Department of Physical Medicine and Rehabilitation, College of Medicine, University of Florida, PO Box 112730, Gainesville, FL 32611 USA

**Keywords:** Back pain, Gait speed, Chair rise, Pain, Quality of life

## Abstract

**Background:**

Patients seeking medical care for back pain often have coexisting painful joints and the effects of different combinations and number of coexisting pain sites (hip, knee, foot/ankle) to back pain on physical function domains and quality of life rating are not yet established. The purpose of this study was to determine the differences in functional outcomes and QOL among individuals with back pain who have concurrent additional pain sites or no pain sites.

**Methods:**

Data from the Osteoarthritis Initiative (OAI) cohort were used for this cross-sectional analysis. Men and women aged 45–79 years with back pain were binned into nine groups by presence or not of coexisting hip, knee, ankle/foot pain and combinations of these sites (*N* = 1,642). Healthy controls reported no joint pain. Main outcomes included Knee Injury and Osteoarthritis Outcome score (KOOS; quality of life and function-sports-and-recreation), Western Ontario McMaster Universities Osteoarthritis Index (WOMAC; Activities of Daily Living, Pain), Medical Outcomes Short Form-12 (SF-12) Physical Component score, and self-reported function in last 7–30 days (lifting 25-pound objects, housework). 20-m and 400-m walk times and gait speed and repeated chair rise test times were collected.

**Results:**

Compared to back pain alone, pain at all five sites was associated with 39%—86% worse KOOS, WOMAC, and SF-12 scores (*p* < .0001). Back-Hip and Back-Knee did not produce worse scores than Back pain alone, but Back-Hip-Knee and Back-Knee-Ankle/Foot did. The 20-m, 400-m walk, and repeated chair times were worse among individuals with pain at all five sites. Additional hip and knee sites to back pain, but not ankle/foot, worsened performance-based walk times and chair rise scores.

**Conclusions:**

The number and type of coexistent lower body musculoskeletal pain among patients with back pain may be associated with perceived and performance-based assessments. Management plans that efficiently simultaneously address back and additional coexistent pain sites may maximize treatment functional benefits, address patient functional goals in life and mitigate disability.

**Supplementary Information:**

The online version contains supplementary material available at 10.1186/s12891-024-07393-2.

## Introduction

Back pain is the most common musculoskeletal problem globally, with the estimated prevalence reaching 577 million in 2017 [1]. Middle-aged and older adults suffer a disproportionate loss of disability-adjusted life years relative to the years lived with back pain [2]. In the United States, up to 33.6% of adults report back pain symptoms during the past three months [3]. Persons with back pain can also experience pain at other sites, and multisite pain occurs more often than single site pain [4]. Localized back pain impairs mobility, activities of daily living and engagement in work [5]. At our institution’s Comprehensive Spine Center, we reported that among patients being treated for back pain, 17.7% had back pain alone, whereas 30.2% had coexistent foot/ankle pain, 46% had hip pain, 44% had knee pain [6]. The number and location of coexisting pain sites along the lower extremity (from foot/ankle to the hip) may differentially worsen specific types of functional impairments such as joint range of motion, movement speed, and mobility, but this has not yet been systematically investigated.

The chronic pain experience among individuals with chronic low back pain in the context of other painful joints is only partially understood. This with back pain are more likely to have pain at other site and widespread pain compared to those without, and painful movement may be a mediator of worse functional health outcomes [7]. Relationships have been proposed among central pain sensitization, pain catastrophizing, self-efficacy, fear evoked movement changes and biopsychosocial phenomena such as somatosensory integration that may contribute to physical function performance [7, 8]. Some investigations focused on overall pain site number rather than specific sites, and found that four pain sites increased the odds of loss of functional independence with walking, climbing stairs, bending, reaching and grasping small objects and reduction of grip strength and gait speed compared to one painful site [9]. Progressively greater numbers of musculoskeletal pain sites has been shown to increase the risk of disability onset in older persons by two-fold [10] and retirement disability pensions over tenfold [11, 12]. Pain in varying musculoskeletal sites (back, foot and elbow) is associated with greater pain severity in other joints like the knee, suggesting centrally-mediated interrelationships between pan site combinations on outcomes [13]. Importantly, subjective data also show that the presence of back pain can also worsen functional and pain scores of osteoarthritis-specific instruments for knee and hip, supporting a potential psychological link and among pain types on these functional outcomes [14–16].

Some studies show that when knee or hip pain are coupled with low back pain, subjective quality of life (QOL) and physical tests are worse than with back pain alone [17, 18], whereas other data do not [19]. A more systematic approach of comparing back pain alone to back pain with different combinations of coexisting lower body joint pain is needed to clearly quantify differences in functionality, work participation, housework chores, lifting objects, self-reported general well-being and disease-specific domains to obtain a true assessment of pain impact in life. Currently, clinical guidelines for management chronic low back pain are comprised of 15 areas, among which include diagnostic education, medications, exercise therapy, and psychosocial interventions only if any factors are identified [20]. Determination of co-existing pain was not described as part of the recommended approach, despite the strong effect on health burden and physical function outcomes for this population.

The publicly-available Osteoarthritis Initiative (OAI) dataset offers a unique opportunity to delineate the impact of specific, coexisting lower body combinations of pain sites in individuals with back pain on multiple domains of physical function and related QOL. As part of this study, the population-based OAI prospectively collected subjective and objective functional outcomes in individuals with different presentations of joint pain including back, knee, hip and foot/ankle. The purpose of this study was to determine the differences in functional outcomes and QOL among individuals with back pain who have concurrent additional pain sites or no pain sites. We hypothesized that patients with back pain combined with foot/ankle pain and knee pain would experience worse outcomes than back pain alone, and that multiple lower body pain sites would amplify mobility limitations compared to one or two additional sites. The study findings could help contribute to future clinical care updates for the management of low back pain with respect to the diagnostic and clinical exam approach to assess other pain sites, wider use of psychosocial interventions based on pain burden beyond spine, and specific rehabilitation exercise content to accommodate more than back pain alone.

## Methods

### Study design

Data were obtained from the OAI clinical dataset version 0.2.3, (release created 9.0401M4). These data are available for public access (https://nda.nih.gov/oai/) The OAI is a multicenter, observational population-based study of knee OA and is comprised of three subgroups (*N* = 4,796): the Incidence Cohort, the Progression Cohort and the Control group. The OAI study protocol, amendments, and informed consent documentation were reviewed and approved by the local institutional review boards of the University of Maryland School of Medicine, Baltimore, The Ohio State University, Columbus, University of Pittsburgh, Memorial Hospital of Rhode Island, Pawtucket and the University of California, San Francisco. Enrolled participants spanned the spectrum of joint health. The Incidence Cohort was comprised of individuals who had OA risks (radiographic and or symptomatic manifestations of OA, but not yet an diagnosis yet). The Progression Cohort consisted of individuals who had diagnoses of OA that were progressing over time. The Control group cohort was comprised of individuals who had healthy knee joints as the comparator in the OAI study design. Irrespective of group or cohort, enrollees could have other co-existent lower joint pains that were not in the knee, hip at the time of enrollment such as foot and ankle.

As originally designed, age-eligible women and men were recruited from the community and enrolled at four recruitment centers (BLINDED FOR REVIEW). The OAI was designed by the original investigators to prospectively observe people over time to better understand the knee osteoarthritis disease process and treatment impact in people at risk for or with the disease. However, the study also captured a variety of pain measures and osteoarthritis diagnoses at other joints including the back, neck and hip among others. As such, this analysis here represents a cross sectional examination of outcomes by pain site at baseline.

### Participants

Participants who reported the following pain patterns were included in this analysis: No Pain, Back pain alone, Back-Hip, Back-Knee, Back-ankle/foot, Back-Hip-Knee, Back-Knee-Ankle/Foot, Back-Hip-Ankle/Foot, and All sites. The choice for these combinations of pain sites was informed by patient pain joint patterns documented in our long-standing institutional comprehensive spine program [6]. We systematically added sites to back to create different joint pain combinations for a total of nine groups. Symptomatic joint pain was defined as: 1) participant report of frequent joint symptoms (hip, knee, ankle, foot) defined as “pain, aching, or stiffness in and around the knee on most days” for ≥ 1 month during the past 12 months, or 2) participant report of having “Any back pain in the past 30 days” [13]. For patients reporting pain in the last 30 days, estimates of frequency of symptoms were provided by the following questions: How often were you bothered by back pain ? (answer choices were rarely, sometimes, most of the time, all of the time) and How bad was the pain? (answer choices were mild, moderate or severe). A total of 25 participants’ pain data from one or more sites were missing from the system. To assure that participants were characterized only with the pain groupings outlined for this analysis, we removed all other participants who had various other pain permutations that could confound the analysis. Thus, a total of 1,642 patients 45–79 years of age were included. Confidentiality has been maintained in this dataset through deidentification by the original investigators and coding to prevent linkage of data to any individual patient.

### Health status at baseline

Anthropometrics, demographics, cigarette smoking status and pain medication use (“taken any pain medication today [include both prescription and over-the-counter medications for any type of pain]”) represented baseline health status. Body mass index (BMI) was calculated as weight (kg) divided by the square of height (m^2^). The age-adjusted Charlson Index was calculated as a comorbid burden estimate, where age adjustment consisted of assigning each decade of life a comorbidity score of one point in addition to presence or not of 19 conditions [21]. Bilateral status of knee, hip and ankle/foot pain was categorized.

### Covariates

Several characteristics were extracted from the datasets that could influence study outcomes. These included educational status (stratified into less than high school, high school graduate, some college or college graduate, some graduate school or completed graduate school), household income (< or ≥ $50,000), and home living situation (living alone or not). This income threshold was selected as it represents a threshold separating lower from higher socioeconomic status for individuals living with chronic pain [22].

### Subjective outcome measures

Several subjective and objective functional metrics were selected to represent functional capacity in the home, work, community and social domains.

#### Knee Injury and Osteoarthritis Outcome Score (KOOS)

The KOOS evaluates short and long-term symptoms in individuals with knee osteoarthritis or injury [23]. Low back pain and widespread joint progressively worsen KOOS scores and is reflective of greater systemic pain burden on physical function [16]. This is a valid and reliable instrument that consists of five different subscales, two of which were provided here: Function in Sports and Recreation (KOOS SFR; involves questions about difficulty with tasks such as jumping, running, squatting or kneeling) and the knee-related quality of life (KOOS QOL; involves questions about awareness of knee problems, modifications to lifestyle). KOOS = Knee Osteoarthritis Outcomes Score [QOL = quality of life; FSR = function, sports and recreation] Scores are percentages ranging from 0 to 100, where 0 = extreme symptoms and 100 = no symptoms [24]. The minimal detectable changes in people with OA are 21.1 points for QOL 21.1 and 19.6 for FSR [25]. Internal consistency for these KOOS subscales ranges from 0.71–0.98 [25].

#### Western Ontario and McMaster Universities Osteoarthritis Index (WOMAC)

The WOMAC instrument is a 24-item questionnaire comprised of three subscales for knee pain and function: pain, stiffness, and physical function [26]. The physical function subscale consists of 17 items relating to activities of daily living (ADL; i.e. rising from sitting, standing, walking, dressing, transfers). Each item is scored from 0–4, where 0 = no difficulty and 4 = extremely difficult. Higher scores indicate greater functional limitation. The WOMAC captures more impact than from just knee or hip pain and dysfunction, and affected by low back pain [14]. Previous studies have found that back pain can increase WOMAC subscores among people with knee OA [14, 15].

#### Physical Activity Score in the Elderly (PASE)

The PASE can provide insight into factors that moderate physical activity among individuals with disabling condition, such as chronic pain. The PASE a valid, 12-item self-administered questionnaire developed for the use in adults over 65 years of age to estimate the amount of physical activity performed over the last seven days [27]. Physical activities include walking outside, household chores (light or heavy), sports and recreational activities (light, moderate and strenuous) and work hours. The intensity level, duration and frequency are used to create a score ranging from 0 to 793 points, with higher scores representing higher activity levels [27]. This instrument has good test–retest reliability (*r* = 0.75) [28] and corresponds with performance based tests such as walk tests and gait speed, joint pain and perceived difficulty with physical function [29].

#### Short form SF-12

The SF-12 instrument captures self-reported impact of health on everyday life [30]. Eight health-related domains with one or two questions per domain are included (limitations in physical activities, social activities and usual role activities, bodily pain, general mental health, vitality and general health perceptions). A Physical Composite Score (PCS) was determined using scores from the 12 twelve questions that ranges from 0–100, where 0 = lowest level of health and 100 = highest level of health. A minimally clinically important difference in SF-12 PCS scores is considered more than 3.29 points among patients with subacute and chronic low back pain [31]. The SF-12 is widely used to measure quality of life among individuals with low back pain and multisite musculoskeletal pain [32–34].

#### Housework and work participation time

Participation in housework and lifting objects by hand were collected in binary and Likert responses. Participant responses to “Lift or move objects weighing 25-pounds or more by hand during single day, past 30 days” was recorded as ‘yes’ or ‘no’ and “How often lift or move objects weighing 25-pounds or more by hand during a typical week, past 30 days” was reported as days per week. Household activity was reported using the items: “Household activities: light housework, part 7 days” and “Household activities: heavy housework, past 7 days,” and vocational work was quantified using the following two items: “When worked how many hours per week usually work, past 12 months (include any overtime hours usually worked)”, and “About how many weeks worked, past 12 months (include paid vacation weeks as weeks worked).”

### Objective functional measures

Objective, valid measures of physical function extracted from the dataset included the repeated chair rise test (timed completion of five chair stands), the 20-m walk test (time to completion of 20-m), and the 400-m walk test (time to completion of the distance, and whether a cane was used for assistance to complete the test, and whether rest stops were needed to complete the test)*.* Long 400-m walk test times are associated with greater mobility limitations, disability and higher mortality rates [35]. Gait speeds are lower among persons with back pain compared to persons without, and gait speed loss is related to loss of independence and quality of life [36]. We determined gait speed by dividing the walk test distance by the time required to complete the walk. Gait speed is expressed in m/s. Gait speed of less than 1 m/s is associated with elevated risk for self-reported mobility disability among older adults [37].

### Statistical analyses

Statistics were conducted in IBM SPSS version 28.0 (Armonk, NY). Normality of distributions were first examined for each outcome variable. For outcomes where skewness and kurtosis existed (weeks worked in last year, 400-m walk time, 20-m walk time and repeated chair rise time, SF-12 Physical subscore), Log10 transformations were performed. Given that the analyses of the transformed variables yielded the same results as the raw data, we present the raw data here. Univariate analyses of variance were applied to address the study aim. Functional outcomes and quality of life outcome scores were the dependent variables (KOOS, WOMAC, PASE, work hours, walk times, chair rise times, gait speeds, SF-12 scores) and the pain site groups were the independent variable. Scheffé post-hoc tests were used to determine where pain site group differences existed. Covariates included sex, age, body mass index, smoking, marital status, education level, home living situation (living alone or not) household income, bilateral joint pain status (for hip, knee, ankle/foot) and Charlson Index. Kruskal–Wallis tests were used to determine whether differences existed among groups for categorical variables (lifting 25-pound weights, doing light and heavy housework, falls, cane use and stops during the 400-m walking test). Logistic regression was performed to determine the Odds Ratio (OR) of presence of mobility disability (yes/ no) among the different pain groups. Models were adjusted for the same covariates as for the univariate analyses described above. Significance was established at *p* < 0.05 for all tests.

## Results

### Participant characteristics

Table 1 provides the characteristics of the study groups. More pain sites were associated with higher BMI values, female sex and use of pain medication (all *p* < 0.05). Supplemental Table 1 provides additional characterization of social determinants of health of the study groups. Participants with all pain sites reported lowest working rates, highest proportion of annual income < $50,000 and not being married, and living alone (all *p* < 0.05).

**Table 1:** Characteristics of the pain groups. Values are means ± SD or percent

	No pain	Back alone	Back-Hip	Back-Knee	Back-Ankle/ Foot	Back-Hip- Knee	Back-Knee Ankle/Foot	Back-Hip Ankle/Foot	All
	*n* = 1109	*n* = 45	*n* = 127	*n* = 78	*n* = 10	*n* = 177	*n* = 20	*n* = 33	*n* = 43
Age (yr)	61.6 ± 9.2	60.6 ± 9.5	61.3 ± 9.2	60.4 ± 9.8	64.1 ± 7.4	60.5 ± 8.8	60.4 ± 9.0	61.1 ± 8.8	60.1 ± 9.2
Weight (kg)	79.5 ± 16.1*	81.2 ± 17.4	78.9 ± 15.4	82.6 ± 14.5	73.0 ± 12.9	82.8 ± 16.7	89.4 ± 18.0	82.6 ± 18.0	89.5 ± 16.3
BMI (kg/m^2^)	27.7 ± 4.4^*^	28.7 ± 5.4^*^	29.0 ± 4.8^*^	28.7 ± 4.9^*^	27.9 ± 5.2	29.4 ± 5.6	31.3 ± 5.5	29.6 ± 4.8	32.8 ± 5.5
Women (#, %)	572 (51.5)	26 (57.8)	97 (76.4)	38 (48.7)	9 (90.0)	116 (65.5)	10 (50.0)	20 (60.6)	34 (79.1)
Race (#, %)									
Black	202 (18.2)	9 (20.0)	22 (17.3)	10 (12.8)	5 (50.0)	27 (15.3)	4 (20.0)	3 (8.9)	11 (25.6)
Caucasian	878 (79.2)	36 (80.0)	101 (79.5)	65 (83.3)	4 (40.0)	147 (83.0)	16 (80.0)	30 (90.1)	31 (72.1)
Other	29 (2.6)	0 (0)	4 (3.2)	3 (3.9)	1 (10.0)	3 (1.7)	0 (0)	0 (0)	1 (2.3)
Ethnicity (#, %)^€^									
Hispanic	14 (1.3)	0 (0)	3 (2.4)	4 (5.1)*	1 (10.0)	1 (0.6)	0 (0)	0 (0)	1 (2.3)
Charlson Index (pts)	2.0 ± 1.3	1.9 ± 1.3	2.0 ± 1.3	1.9 ± 1.3	2.1 ± 0.7	1.9 ± 1.1	1.9 ± 1.2	1.8 ± 1.1	2.0 ± 1.5
Back pain symptoms (#, %)									
How often bothered in last 30 days?^€^									
Rarely	861 (77.6)	0 (0)	0 (0)	0 (0)	0 (0)	0 (0)	0 (0)	1 (3.0)	0 (0)
Sometimes	227 (20.4)	0 (0)	0 (0)	0 (0)	0 (0)	0 (0)	0 (0)	0 (0)	0 (0)
Most times	21 (2.0)	25 (55.6)	84 (66.1)	47 (60.3)	8 (80.0)	107 (60.5)	13 (65.0)	18 (54.5)	25 (58.1)
All the time	0 (0)	20 (44.4)	43 (33.9)	31 (39.7)	2 (20.0)	70 (39.5)	7 (35.0)	14 (42.4)	18 (41.9)
How bad was the pain over last 30 days?									
Mild	322 (29.0)	0 (0)	67 (46.5)	0 (0)	0 (0)	0 (0)	0 (0)	0 (0)	0 (0)
Moderate	112 (10.1)	38 (84.4)	62 (43.1)	107 (84.3)	9 (90.0)	128 (72.3)	17 (85.0)	26 (78.8)	29 (67.4)
Severe	11 (1.0)	7 (15.6)	15 (10.4)	20 (15.7)	1 (10.0)	49 (27.7)	3 (15.0)	6 (18.2)	14 (32.6)
Bilateral (%)	----	-----	31.4	64.1	80.0	37.8 (hip)	70.0 (knee)	42.4 (hip)	60.5 (hip)
						70.6 (knee)	50.0 (ankle/foot)	54.5 (ankle/foot)	83.7 (knee)
									100 (ankle/ foot)
Taken pain medication today? (#, %)^€^									
	83 (7.5)	5 (11.1)	18 (14.2)	14 (17.9)	2 (20.0)	45 (25.4)	4 (20.0)	10 (30.3)	9 (20.9)

^*^differs from All Sites at *p* < .05

^€^significant $${x}^{2}$$ statistic *p* < .05

### Outcome measures

The self-reported KOOS, WOMAC activities of daily living subscore, PASE, SF-12 and work volume performed in the last year are presented in Table 2. More pain sites were associated with worse KOOS QOL and FSR scores than no pain or back pain alone (*p* < 0.0001). Similarly, more pain sites were associated with worse WOMAC activities of daily living score than no pain or back pain alone (*p* < 0.0001). SF-12 Physical scores were worse with additional pain sites to back pain alone (*p* < 0.0001). Total PASE scores and work weeks and hours worked per week were not different among the pain site groups.

**Table 2:** Outcome measures by pain site grouping. Values are means ± SD

	No pain	Back alone	Back-Hip	Back-Knee	Back-Ankle/Foot	Back-Hip-Knee	Back-Knee Ankle/Foot	Back-Hip Ankle/Foot	All sites
	*n* = 1109	*n* = 45	*n* = 127	*n* = 78	*n* = 10	*n* = 177	*n* = 20	*n* = 33	*n* = 43
**KOOS scores (points)**									
QOL	80.9 ± 18.2^^^	71.1 ± 19.9	68.9 ± 22.1	65.1 ± 19.4	59.4 ± 39.0	53.5 ± 19.5^*€^	57.1 ± 23.4^* Ψ^	64.0 ± 24.5	49.7 ± 25.8^*€**^
FSR	85.8 ± 18.8^	73.2 ± 25.3	67.0 ± 25.8^**^	66.8 ± 24.9	65.4 ± 33.4	50.9 ± 24.9^*€^	53.7 ± 30.7*	68.9 ± 23.8	44.7 ± 30.0^*€**^
**WOMAC score (points)**									
Pain	1.1 ± 2.0^^^	2.3 ± 2.9^^^	2.9 ± 3.4^¥^	2.9 ± 3.1^*Ψ^	4.5 ± 3.6^*^	4.1 ± 3.9^^^	3.4 ± 3.8^^^	3.3 ± 4.6^^^	7.0 ± 6.1^^^
ADL	3.6 ± 5.8^^^	9.4 ± 12.0^¥**^	9.8 ± 10.4^¥^	11.2 ± 11.2^^Ψ^	11.9 ± 10.3^¥^	15.9 ± 12.7^*^	15.2 ± 12.1	13.1 ± 14.5^* Ψ^	25.4 ± 16.1^^^
**SF-12 scores (points)**									
PCS	52.8 ± 6.8^^^	46.9 ± 9.8^^**^	46.0 ± 10.7^¥^	45.4 ± 10.0	43.3 ± 17.8^**^	42.6 ± 11.1^δ * Ψ^	43.3 ± 11.5^δ^	42.7 ± 10.0^δ^	39.7 ± 9.5^* Ψ^
**Working in past year**									
Weeks (#)	46.0 ± 12.6	48.8 ± 7.8	47.3 ± 10.6	46.7 ± 10.6	41.6 ± 18.7	46.2 ± 12.0	45.9 ± 12.1	43.9 ± 16.1	46.5 ± 11.5
Hours/week (#)	36.2 ± 15.2	34.3 ± 15.1	37.8 ± 15.1	37.6 ± 13.2	27.5 ± 14.2	38.0 ± 15.8	32.6 ± 14.6	36.2 ± 17.0	29.2 ± 17.1

*QOL* quality of life, *FSR* Function, Sports and Recreation, *ADL* activities of daily living, *PCS* physical component score

^^^different than all other groups at *p* < .05

^δ^different than No pain group at *p* < .05

^*^different than Back alone and No pain groups at *p* < .05

^Ψ^different than hip-back at *p* < .05

^**^different than Back-Hip-Ankle/foot groups at *p* < .05

^€^different than Back-Hip and Back-Knee groups at *p* < .05

^¥^different than No pain, Back alone, Back-Knee, Back-Hip, Back-Hip-Knee, at *p* < .05

**Fig. 1:**
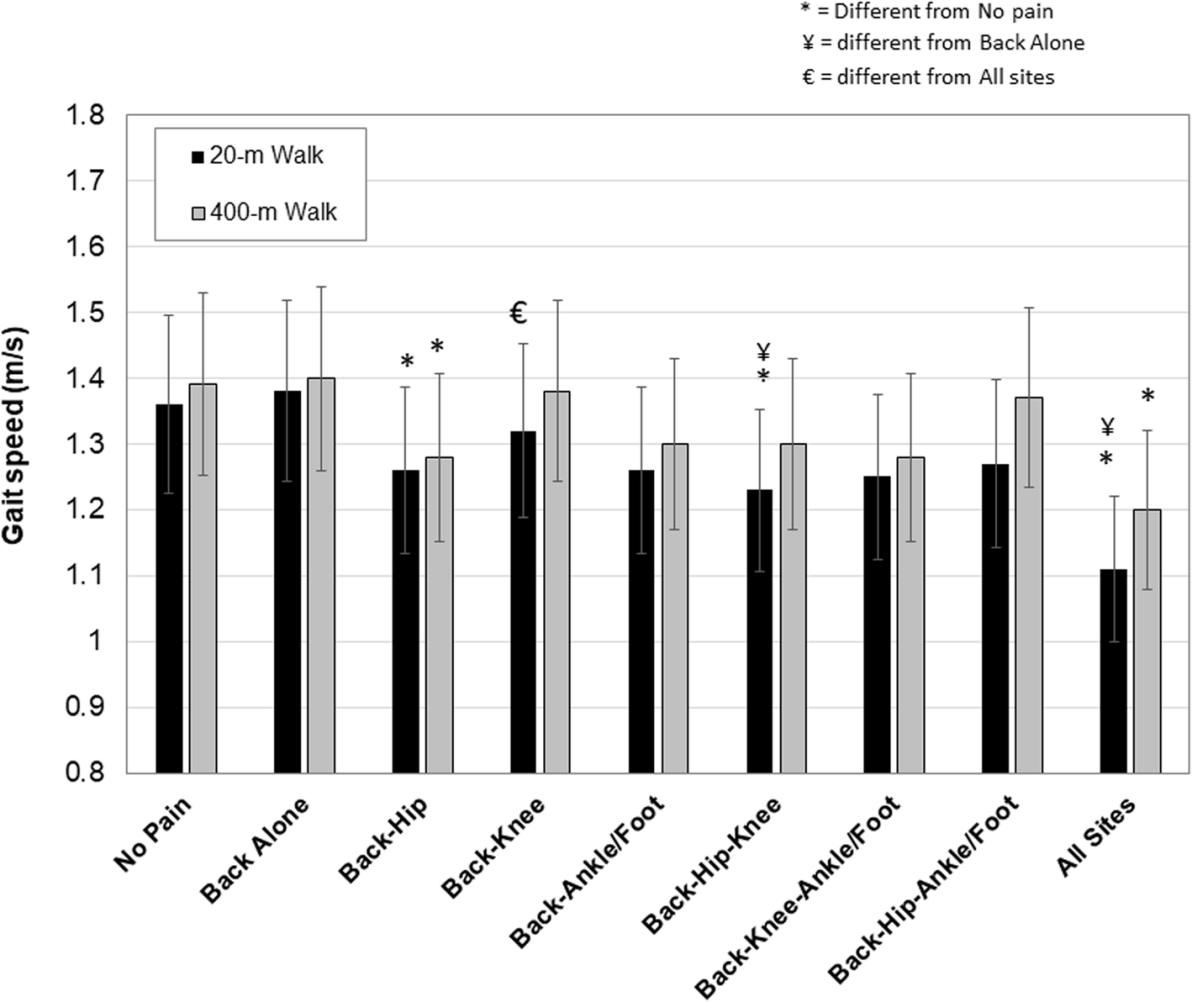
Gait speeds achieved in the 20-m and 400-m walk tests. Means and standard deviations (SD) are shown

Subjective and objective functional performance measures are reported in Table 3. Participants with multiple pain sites reported lifting 25-pound objects by hand in a single day over the last 30 days less often than participants with back pain alone (*p* < 0.01). There were no differences among the groups for reporting engagement in light or heavy housework or lifting 25-pound objects by hand in the last seven days. In general, additional pain sites were associated with longer times to complete the 20-m walk test, 400-m walk test and repeated chair rise test (all *p* < 0.001). Gait speeds for the 20-m and 400-m walk tests are shown in Fig. 1. The No Pain group demonstrated faster gait speeds for both walk tests than nearly all combinations of pain (*p* < 0.05). Different combinations of pain sites with back pain impacted gait speeds compared to the No pain group, particularly for the 20-m walk.

**Table 3:** Performance of functional activities and functional test scores based on pain sites. Values are means ± SD (95% CI) or expressed as number and proportion of the group who indicated an answer of “yes”

	No pain	Back alone	Back-Hip	Back-Knee	Back-Ankle/Foot	Back-Hip-Knee	Back-Knee Ankle/Foot	Back-Hip Ankle/Foot	All sites
	*n* = 1109	*n* = 45	*n* = 127	*n* = 78	*n* = 10	*n* = 177	*n* = 20	*n* = 33	*n* = 43
**Self-Reported Function**									
Lifted objects by hand 25 lbs or more in single day, last 30 days (#, %)									
	824 (74.3)	36 (80.0)	80 (62.9)	46 (59.0)	7 (70.0)	114 (64.4)	13 (65.0)	20 (60.6)	28 (65.1)
Days lifted 25 lb objects in last 7 days (# yes, %)									
0 -1 days	605 (54.1)	19 (56.2)	72 (61.1)	41 (45.3)	2 (20.0)	98 (55.4)	11 (55.0)	19 (57.6)	21 48.8)
2–5 days	385 (35.3)	20 (32.9)	41 (30.6)	24 (40.0)	5 (50.0)	60 (33.9)	5 (25.0)	10 (30.3)	17 (39.5)
Near all/all days	116 (10.6)	5 (10.5)	13 (8.3)	12 (13.2)	3 (30.0)	17 (9.6)	4 (20.0)	4 (12.1)	5 (11.6)
Performed light housework in last 7 days (# yes, %)									
	1024 (92.3)	44 (97.8)	117 (92.1)	73 (93.6)	10 (100.0)	161 (91.0)	19 (95.0)	29 (87.9)	42 (97.7)
Performed heavy housework in last 7 days (# yes, %)									
	810 (73.0)	34 (75.6)	92 (72.4)	53 (67.9)	6 (60.0)	133 (75.1)	16 (80.0)	24 (72.7)	35 (81.4)
**Performance-Based Function**									
20-meter walk (s)									
	14.9 ± 2.5	14.8 ± 2.6^¥^	16.2 ± 3.1	15.8 ± 4.7	14.9 ± 2.3	16.3 ± 3.6	16.6 ± 3.0	15.7 ± 1.9	19.2 ± 4.7^*^
400-meter walk (s)									
	295 ± 47^Ψ^	294 ± 53	321 ± 70	310 ± 84^€^	319 ± 52	324 ± 72	324 ± 53	313 ± 85	350 ± 78^δ*^
Repeated chair rise time (s)									
	10.4 ± 2.9^¥^	12.1 ± 4.1^δ^	12.4 ± 5.3^δ^	12.2 ± 4.4^δ ^^^	11.5 ± 3.5	13.5 ± 5.7^¥^	13.0 ± 4.6^δ^	12.0 ± 4.4^^^^	13.0 ± 5.3^δ^

^^^different than all other groups at *p* < .05

^δ^different than No pain group at *p* < .05

^*^different than Back alone

^Ψ^different than Back-Hip at *p* < .05

^**^different than Back-Hip-Ankle/Foot groups at *p* < .05

^^^^different than Back-Hip-Knee

^€^different than Back-Hip and Back-Knee groups at *p* < .05

^¥^different than Back alone, Back-Knee, Back-Hip, Back-Hip-Knee, Back-Hip-Ankle/Foot at *p* < .05

### OR for mobility disability

Table 4 provides the OR values (95% confidence interval [CI]) for the presence of mobility disability by pain group. The OR for disability were significantly higher for Back-Hip, Back-Hip-Knee groups and All sites compared to no pain (all *p* < 0.05).

**Table 4:** Odds ratios (OR) for presence of mobility disability (gait speed < 1.0 m/s) with different pain site combinations

	B (95% CI)	*p*
No Pain	reference	
Back Alone	0.68 (0.09 – 5.31)	.713
Back-Hip	2.612 (1.28 – 5.36)	.008
Back-Knee	1.62 (0.52 – 5.09)	.409
Back-Ankle/Foot	2.71 (0.2 – 24.735)	.377
Back-Hip-Knee	3.43 (1.81 – 6.49)	< .001
Back-Knee-Ankle/Foot	3.45 (0.66 – 8.15)	.144
Back-Hip-Ankle/Foot	0.73 (0.94 – 5.97)	.749
All sites	8.36 (3.37 – 20.77)	< .001

## Discussion

We examined the effects of specific, coexisting pain sites and combinations of pain sites in individuals with back pain on several domains of physical function and related quality of life. The coexistence of hip-knee pain with back pain or of pain at all sites was associated with worse self-reported physical function and quality of life ratings (WOMAC, SF-12, KOOS) and 20-m walk test time compared to the combination of back pain with hip or knee pain separately. All pain site group, the presence of foot/ankle pain did not result in worse self-reported or performance based functional scores except for gait speed. The risk for mobility disability was significant among individuals with Back-Hip, Back-Hip-Knee pain and All sites, with the greatest risk in the All sites group with an OR of 8.4. These findings show that both combination and number of lower body pain sites in addition to back pain can differentially impede daily function. From the clinical perspective, the presence of different pain sites among patients with low back pain could help with development of specific functional goals, and improve precision of physical therapy content.

### Objective physical function and work participation

We documented worse repeated chair rise time and walking performance tests, particularly among people with knee and hip pain in addition to back pain. Walking times were significantly longer in the All sites group only, but gait speed was impaired in the Back-Hip, Back-Hip-Knee and All sites groups. In other published studies of patients with different combinations of pain sites, slower gait speed occurred in persons with combined knee-back pain than the no pain or knee pain or back pain groups alone [38]. Older adults with back-hip pain take 25.6% more time to perform repeated chair rises than adults with back pain [18]. Multisite data showed that stair climb time is 17.8% longer in people with multijoint pain than people with back pain alone [16]. Roseen et al. [39] found from the Boston RISE study that older adults with multisite pain had 6.3% slower gait speed, 4.7% worse performance on the short physical performance battery, and 5% lower leg strength than those with single site pain. Unlike other cohort studies that enroll persons across the whole adult age spectrum, the OAI enrolled persons with knee and hip osteoarthritis aged 45–79 years. Potential mechanisms underlying functional impairment with multisite pain (≥ 2 sites) may be pain sensitization [16], fear and kinesiophobia, lower leg strength and speed [39], poor balance-coordination performance [40] and low physical activity levels [41]. Our findings could provide support of standardized collection of co-existing joint pain during history taking in clinical encounters. Depending on the pain site combination and pain site number among patients with back pain, the specific type of therapeutic activities (e.g., land or aquatic type, single or multi-joint activity, weight supported or ambulatory) and progression of therapy (from single to multijoint, intensity and rate of progression) can be better tailored to enhance patient engagement. Moreover, clinicians can identify early which patients with back pain may need additional psychological support to overcome and adapt in response to pain challenges during treatment. Longitudinal interventions are needed to address these important areas.

Pain sites in addition to back pain were not associated with less work participation in the past 12 months. Our findings are in contrast with those of Coggon et al. [42]. These authors prospectively showed that work absence increased with the additional number of sites other than back pain among 47 occupational groups (*N* = 12,426). Widespread international variation exists in the prevalence of disabling musculoskeletal pain among working adults [42]. Other individual factors exist that influence the propensity to pain [42], pain processing (high somatisizing) [43], cultural-behavioral response to pain (expectations of pain tolerance), and nature of recurrence of pain (e.g., frequent, predictable with physical loading, or sporadic). Other factors may be influencing work participation including occupational postures, type of work exposures (long shifts or several short shifts), job strain and social support [4]. With a standardized history taking approach inclusive of other joint pain sites, clinicians may identify some of these specific barriers to work participation and inform therapy and subspecialist treatment targets.

### Subjective physical function and quality of life and life context

We found that subjective KOOS-Function, Recreation and Sport scores and SF-12 Physical Component scores worsened among people with Back-Hip-Knee pain and were worst in the All sites group. Other investigators have reported associations between more pain sites, worse subjective activity limitations [44], more difficulties with social activities, and moderate-to-severe social activity limitations [45]. Iijima et al. also found 42.8% greater difficulty with activities of daily living scores in persons with back-knee pain than knee pain alone [17]. Other evidence shows that lower SF-12 Physical Component scores occur with additional peripheral joint sites [46]. de Luca et al. [47] collected prevalence of pain in the back, neck and peripheral sites as part of the Australian Longitudinal Study on Women’s Health. These authors found that compared to pain-free women, women with ‘some’ joint pain (1–4 sites) and multisite pain (5–22 joints) demonstrated 5% and 24% worse SF-36 Physical Component scores, respectively. A UK population study found that the number of pain sites explained 48% of the variance in a regression model of SF-12 scores among adults age ≥ 50 years [48].

When put into the context of daily living, the magnitude of differences observed in KOOS and SF-12 scores are important for people with Back-Knee-Hip, and All sites of pain. The minimal detectable changes in KOOS QOL and FSR scores are 21.1 points and 19.6 points [25], respectively, but we observed 30–33 point differences among people with no pain to those with four pain sites. Moreover, the differences in SF-12 reveal that persons with back pain and at least two other load-bearing sites in the current study felt a significant loss of life quality due to pain-limited physical function that exceeds the minimal clinically important difference. The KOOS scores also indicated limitations in physical activities that are sports and exercise-related. Thus, physical aspects of life including performance of moderate activities (ex: vacuuming, pushing a table, bowling or playing golf), stairs, housework, vocational activities and participation in exercise and sports are negatively impacted.

### Clinical implications

Pain at one joint site commonly contributes to pain onset elsewhere [40, 49]. Secondary issues related to elevated pain burden include emotional health issues [45], psychological distress [42], depression [40] and risk for other adverse health events like bone fractures [50], cognitive decline and dementias [51]. Chronic pain acts through a cognitive pathway as a distractor to impair function [52], and loss of mobility over time may contribute a feed-forward cycle of cognitive worsening and functional loss. Inclusion of plans for emotional wellbeing may also improve the ability to cope with pain while maintaining or improving functional mobility levels. A more well-rounded approach to back pain care might optimize the patient’s trajectory for functional restoration. With added sub-specialization of healthcare, clinicians are often providing niche medicine that can miss this systemic impact of pain on patients.

Given that more widespread pain negatively impacts walking, chair rise and gait speed, physical therapy could emphasize vertical body transfers with power, walking endurance and trunk and leg conditioning [36]. A kinetic-chain based therapy approach that addresses back pain and co-existent sites through multijoint, complex exercises could be a time-efficient and effective method to improve overall functional capacity rather than back pain alone. Exercise content could also be catered to address goals about specific functional domains that are important to the patient (vocational, occupational, social), rather than follow a generic program for back pain.

### Limitations, strengths and future directions

We acknowledge that the measurement of pain in the OAI (as aching/stiffness and frequency of symptoms) alone may not be a complete representation of osteoarthritis pain at the affected sites. This was a cross sectional analysis and does not provide causation for pain sites and functional and QOL differences observed here. Residual confounding error may exist in this analysis, as we were unable to precisely account for etiology of back pain, retirement status, and potential pain medicine effects on pain severity at the time of the assessments. Response bias may exist from the use of subjective pain assessments. We describe here a snapshot in time and pain at sites other than the back may change over time during the course of medical care. The etiology of pain in the back and neck were not specifically determined in the OAI, and this information might have provided insight on functional and QOL outcomes in this analysis. Functional performance may be differentially impacted by pain etiology, bilateral status and location of the pain.

The OAI is characterized by a high proportion of Caucasians aged 45–79 years and may not be indicative of the broader population of patients typically seen in clinic that provides care for chronic back pain. African-American and Hispanic individuals report higher prevalence of ‘high impact chronic low back pain’, which is related to more severe physical disability compared to people with Caucasian race [39]. Moreover, higher perceived social status is associated with less pain interference and pain outcomes in Causaisans, but not African-Americans [53]. Thus, our findings may under-represent the physical impairment experienced and pain impact by the general population with low back pain. We do acknowledge that the cell sizes of some groups are small and may not represent the general population. Importantly, the pain severity levels at each site may influence these functional outcomes; individuals with high pain severity demonstrate clinically-relevant losses in gait speed compared to low pain severity [38]. Unfortunately, pain severity was not consistently measured across all pain sites in the OAI. Information about the chronicity of pain at each site was also not included in the dataset, which is relevant to progressive and persisting functional changes. The strengths of this study include a large sample size and valid, subjective and objective assessments of physical function. Importantly, this study also enrolled healthy individuals without osteoarthritis as well as varying stages of hip and knee osteoarthritis, and with a spectrum of comorbid conditions. We contend that the sample adequately reflects people with back pain and common coexisting pain sites, and that our findings can be applied to the general back pain population. Future research could address back pain and multisite pain from the patient perspective, using qualitative methods to better understand the real-life impact in daily living and psychological health. Additional avenues of work could directly advance care pathways through investigation of customized interventions to help patients overcome the burden of co-existing multisite pain. Interventions could include combinations of whole body movement therapies, psychosocial approaches and resilience training.

## Conclusion

This cross sectional analysis showed that compared to back pain alone, additional coexistent lower limb musculoskeletal pains, particularly in the hip and/or knee, are negatively associated with physical function and QOL ratings. The risk for mobility disability increases with the addition of Hip pain or Hip-Knee pain to back pain, and is worse when pain is present in all major lower limb joints. Determination of coexisting pain presence and longitudinal pain experience among more diverse patient populations is needed to improve our understanding of pain burden and daily functioning for the general population with chronic low back pain. This evidence is critical to most effectively develop clinical treatment goals and plans that can enhance quality of life.

### Supplementary Information

**Supplementary Material 1.**

**Supplementary Material 2.**

## Data Availability

The datasets used and/or analyzed during the current study are available as a Supplementary file.

## Abbreviations

ADL: Activities of daily living
KOOS: Knee Injury and Osteoarthritis Outcome score
QOL: Quality of life
OAI: Osteoarthritis Initiative
OR: Odds ratio
PASE: Physical Activity Score in the Elderly
SF-12: Medical Outcomes Short Form-12
WOMAC: Western Ontario McMaster Universities Osteoarthritis Index
