# Supplementary material for: Considerations beyond spine pain: do different co-occurring lower body joint pains differentially influence physical function and quality of life ratings?
Source: BMC Musculoskelet Disord. 2024 Apr 8;25:269. doi: 10.1186/s12891-024-07393-2 (PMC11000411; doi:10.1186/s12891-024-07393-2)
Supplement: Supplementary file 1 — Supplementary Material 1. [file 12891_2024_7393_MOESM1_ESM.docx]

Supplemental Table 1. Additional social determinants of health of pain site groups. Values are means ± SD or percent of the group. All χ^2^ analyses for each variable were significant at p<.05

No pain Back alone Back-Hip Back-Knee Back-Ankle/ Back-Hip- Back-Knee Back-Hip All

Foot Knee Ankle/Foot Ankle/Foot

n=1109 n=45 n=127 n=78 n=10 n=177 n=20 n=33 n=43

Working status (#, %)

Working 713 (64.4) 29 (64.4) 69 (55.2) 47 (60.3) 8 (80.0) 97 (54.8) 14 (70.0) 18 (54.5) 21 (48.8)

Not working 30 (2.7) 4 (8.9) 14 (11.2) 8 (10.4) 0 (0) 30 (16.9) 1 (5.0) 5 (12.2) 11 (25.6)

due to health

Not working 365 (32.9) 12 (26.7) 42 (33.6) 22 (28.6) 2 (20.0) 50 (28.2) 5 (25.0) 10 (30.3) 11 (25.6)

other reasons

Missing 1 0 2 1 0 0 0 0 0

Education (#, %)

<high school 27 (2.4) 1 (2.2) 8 (6.3) 4 (5.3) 0 (0) 7 (4.1) 2 (20.0) 1 (3.1) 3 (7.1)

High school

graduate 113 (10.2) 3 (6.7) 14 (11.1) 10 (13.2) 1 (10.0) 28 (16.4) 3 (15.0) 3 (9.4) 8 (19.0)

>high school 965 (87.3) 41 (91.1) 104 (82.5) 62 (81.6) 9 (90.0) 136 (79.5) 15 (75.0) 28 (87.5) 31 (73.8)

education

Missing 4 0 1 2 0 6 0 1 1

Yearly income

< $50,000 343 (33.1) 20 (45.5) 50 (41.7) 28 (39.4) 3 (33.3) 81 (49.7) 8 (44.4) 21 (65.6) 27 (73.0)

> $50,000 693 (66.9) 24 (54.5) 70 (58.3) 43 (60.6) 6 (66.7) 82 (50.3) 10 (55.6) 11 (34.4) 10 (27.0)

Missing 73 1 7 7 1 14 2 1 10

Living Alone (#, %)

228 (20.6) 11 (24.4) 35 (27.6) 17 (21.8) 2 (20.0) 47 (26.6) 8 (40.0) 8 (24.2) 18 (41.9)

Marital Status (#, %)

Married 795 (71.9) 26 (57.8) 78 (61.4) 50 (67.6) 7 (70.0) 94 (54.7) 13 (65.0) 15 (46.9) 14 (34.1)

Widowed 82 (7.4) 2 (4.4) 10 (7.9) 5 (6.8) 1 (10.0) 14 (8.1) 1 (5.0) 7 (21.9) 5 (12.2)

Other 228 (20.6) 17 (37.8) 39 (30.7) 19 (27.5) 2 (20.0) 64 (37.2) 6 (30.0) 10 (31.2) 22 (53.7)

Missing 4 0 0 4 0 5 0 1 2

Smoking now (#, %)

58 (5.2) 5 (11.1) 19 (15.0) 6 (7.7) 0 (0) 20 (11.3) 0 (0) 3 (9.1) 4 (9.3)
